# The effect of modafinil on the rat dopamine transporter and dopamine receptors D1–D3 paralleling cognitive enhancement in the radial arm maze

**DOI:** 10.3389/fnbeh.2015.00215

**Published:** 2015-08-19

**Authors:** Yasemin Karabacak, Sunetra Sase, Yogesh D. Aher, Ajinkya Sase, Sivaprakasam R. Saroja, Ana Cicvaric, Harald Höger, Michael Berger, Vasiliy Bakulev, Harald H. Sitte, Johann Leban, Francisco J. Monje, Gert Lubec

**Affiliations:** ^1^Department of Pharmaceutical Chemistry, University of ViennaVienna, Austria; ^2^Department of Neurophysiology and Neuropharmacology, Center for Physiology and Pharmacology, Medical University of ViennaVienna, Austria; ^3^Core Unit of Biomedical Research, Division of Laboratory Animal Science and Genetics, Medical University of Vienna, HimbergAustria; ^4^Center of Brain Research, Medical University of ViennaVienna, Austria; ^5^TOSLab, Ural Federal University, YekaterinburgRussia; ^6^Institute of Pharmacology, Center of Physiology and Pharmacology, Medical University of ViennaVienna, Austria

**Keywords:** modafinil, radial arm maze, BN-PAGE, working memory, dopamine receptor, dopamine transporter

## Abstract

A series of drugs have been reported to increase memory performance modulating the dopaminergic system and herein modafinil was tested for its working memory (WM) enhancing properties. Reuptake inhibition of dopamine, serotonin (SERT) and norepinephrine (NET) by modafinil was tested. Sixty male Sprague–Dawley rats were divided into six groups (modafinil-treated 1–5–10 mg/kg body weight, trained and untrained and vehicle treated trained and untrained rats; daily injected intraperitoneally for a period of 10 days) and tested in a radial arm maze (RAM), a paradigm for testing spatial WM. Hippocampi were taken 6 h following the last day of training and complexes containing the unphosphorylated or phosphorylated dopamine transporter (DAT-CC and pDAT-CC) and complexes containing the D1–3 dopamine receptor subunits (D1–D3-CC) were determined. Modafinil was binding to the DAT but insignificantly to SERT or NET and dopamine reuptake was blocked specifically (IC_50_ = 11.11 μM; SERT 1547 μM; NET 182 μM). From day 8 (day 9 for 1 mg/kg body weight) modafinil was decreasing WM errors (WMEs) in the RAM significantly and remarkably at all doses tested as compared to the vehicle controls. WMEs were linked to the D2R-CC and the pDAT-CC. pDAT and D1–D3-CC levels were modulated significantly and modafinil was shown to enhance spatial WM in the rat in a well-documented paradigm at all the three doses and dopamine reuptake inhibition with subsequent modulation of D1–3-CC is proposed as a possible mechanism of action.

## Introduction

Cognitive enhancement (CE) is one of the major concerns as cognitive impairment is a hallmark of aging and several brain disorders. Several drugs are available for the treatment of cognitive impairment and modafinil 2-[(diphenylmethyl) sulfinyl] acetamide is one recommended and prescribed recently for CE (Farah et al., 2004; Sofuoglu et al., 2013) due to relatively low adverse effects (Hermant et al., 1991).

Several studies have shown improved memory performance in modafinil-treated rodents: Béracochea et al. (2001, 2003) reported that modafinil (64 mg/kg) improves delay-dependent working memory (WM) in mice and subsequently improvement of WM in serial spatial discrimination reversal T-maze was demonstrated. Modafinil also improved performance of spatial memory in a Morris water maze (75 mg/kg) and fear memory using contextual fear conditioning (0.75 mg/kg; Shuman et al., 2009). Piérard et al. (2006) observed that modafinil led to optimal WM performance in a T-maze at the dose of 8 mg/kg. Eagle et al. (2007) revealed dose-dependent CE effects, i.e., rats performed better in a stop-signal reaction time task at 3 and 10 mg/kg than at doses of 30 or 100 mg/kg.

Cognitive enhancement effects of modafinil have been studied extensively in human volunteers: modafinil improved performance in short-term memory, WM, and inhibition control in sleeping disorders (Scoriels et al., 2013). In attention deficit hyperactivity disorder, modafinil exhibited positive effects on short- and long-term memory (Turner et al., 2004a,b). Studies using modafinil on memory deficits in schizophrenic patients are quite promising and improvement of short-term verbal memory, verbal WM performance, spatial WM errors (WMEs) and strategy use is furthermore observed in schizophrenics (Turner et al., 2004a,b; Scoriels et al., 2012). Minzenberg et al. (2014) studied modafinil effects in schizophrenia and observed improvement of rule selection and representation. Moreover, modafinil was studied in healthy volunteers and significantly enhanced performance in trials of cognition such as WM, cognitive flexibility, and planning (Muller et al., 2004; Turner et al., 2004a,b; Finke et al., 2010).

As to probable mechanisms of actions, modafinil binds to the dopamine transporter (DAT) increasing synaptic dopamine levels exciting dopaminergic and adrenergic receptors resulting in wakefulness (Wisor, 2013). Following modafinil administration hippocampal excitatory glutamatergic neurotransmission increased and GABAergic neurotransmission was decreased (Ferraro et al., 1997). Own previous results indicated that GluA1 and GluA2-containing receptor complex levels were increased in trained drug treated mice (Sase et al., 2012).

Although there is vast evidence for involvement of the dopaminergic system including receptors and DAT, there is limited information on the assembly of DAT and pDAT, D1R, D2R, and D3R-containing complexes (CC’s) but rather than on transporter and receptor subunits.

It was therefore the aim of the study to show cognitive enhancing effects of modafinil at three different doses and hippocampal dopamine transporter and receptor complex levels paralleling WMEs in the RAM and indeed, D2R and pDAT-CC’s were linked to WM.

## Materials and Methods

### Synthesis of Modafinil

Modafinil was synthetized according to a published method (Chatterjie et al., 2004; Rebiere et al., 2010).

### Uptake and Release Assays

Dulbecco’s modified Eagle’s medium (DMEM) and trypsin were purchased from PAA Laboratories GmbH (Pasching, Austria). Fetal bovine serum was purchased from Invitrogen. [3H] 5-HT ([3H] 5-hydroxytryptamine; [3H] serotonin; 28.3 μCi/mmol) and [3H] DA ([3H]dihydroxyphenylethylamine, [3H]dopamine; 46 μCi/mmol) were purchased from Perkin Elmer, Boston, MA, USA. [3H]1-Methyl-4-phenylpyridinium ([3H]MPP+; 85 μCi/mmol) was supplied by American Radiolabeled Chemicals (St. Louis, MO, USA). Paroxetine was purchased from Santa Cruz Biotechnology, USA, while mazindole and D-amphetamine were purchased from Sigma–Aldrich Co.

For uptake experiments, the human isoforms of DAT, SERT, and NET were expressed in HEK293 (HEK-DAT, HEK-SERT, and HEK-NET) cells. Modafinil-mediated monoamine transporter effects on substrate uptake were analyzed as described previously (Sucic et al., 2010). In brief, cells were grown in poly-d-lysine (PDL) coated 96-well plates. Modafinil was dissolved in DMSO and subsequently diluted in Krebs–Ringer–HEPES buffer (KHB; 25 mM HEPES.NaOH, pH 7.4, 120 mM NaCl, 5 mM KCl, 1.2 mM CaCl_2_, and 1.2 mM MgSO_4_ supplemented with 5 mM D-glucose). To determine unspecific uptake in HEK-DAT and HEK-NET 10 μM of mazindole were used while 10 μM of paroxetine were used for HEK-SERT. The tritiated substrates used to assess transport activity at HEK-DAT, HEK-SERT, and HEK-NET, were 0.2 μM ^3^H-DA, 0.4 μM ^3^H-5HT, and 0.05 μM ^3^H-MPP^+^, respectively. Cells were washed once with KHB buffer and incubated with compounds either 5 min for HEK-DAT and HEK-SERT cells or 8 min for HEK-NET cells. Subsequently, substrates were added and the reactions were stopped with ice-cold KHB buffer after either 1 min for HEK-DAT and HEK-SERT cells or 3 min for HEK-NET cells. Cells were lysed with 1% SDS and released radioactivity was measured by a liquid scintillation counter (Tri-carb-2300TR, Perkin Elmer). All the experiments were repeated in triplicate.

**FIGURE 1:**
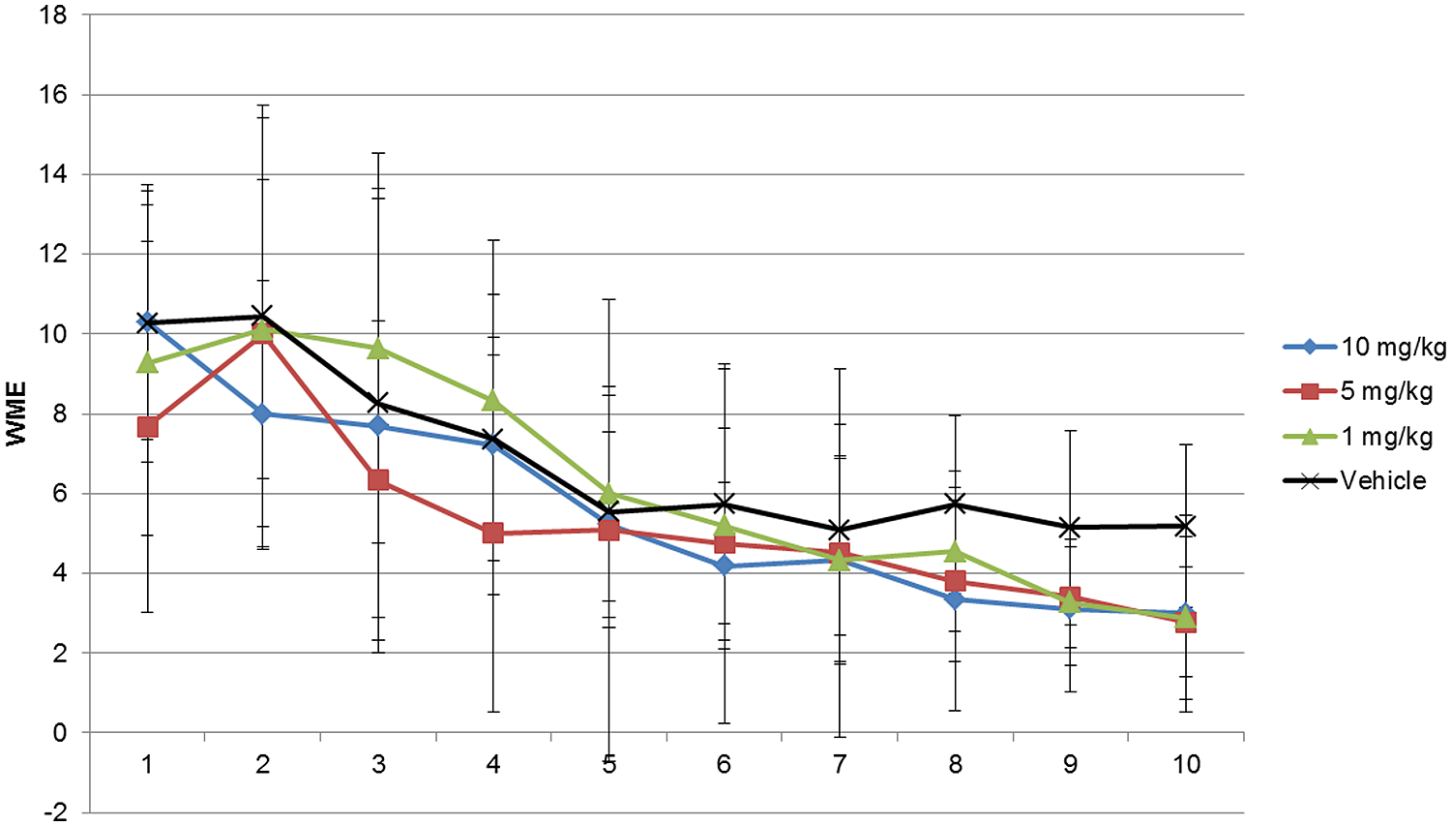
**Working memory error (WME) assessment of modafinil in the radial maze for 10 days**. Rats in groups of 10 and 5 mg/kg body weight modafinil performed at a comparable level in the radial arm maze (RAM) training up to day 7, but from day 8 trained drug animals performed better as compared with their counterparts injected with vehicle whereas rats in groups of 1 mg/kg body weight performed better on day 9 and 10. Data analyzed using ANOVA and Bonferroni *post ho*c test. Data represented as mean ± SD.

The substrate/efflux experiments were performed as described before (Sucic et al., 2010). Briefly, HEK-DAT cells were grown in 5 mm diameter PDL-coated coverslips. Cells were incubated with 0.1 μM ^3^H-MPP^+^ at 37°C for 20 min. The coverslips were transferred onto superfusion chambers (0.2 ml) and excess radioactivity was washed out with KHB buffer for 40 min (0.7 ml/min) at 25°C to obtain stable baselines. Thereafter, modafinil or D-amphetamine was added as depicted in **Figure 1** and the experiment was started with the collection of fractions (2 min). During the experiments with enhanced intracellular sodium concentration, the buffer was switched either to monensin or remained at control buffer after the collection of three baseline fractions for another five fractions. Subsequently, modafinil or D-amphetamine was added for another five fractions as indicated in **Figure 1**. Finally, the remaining radioactivity was collected by treatment with 1% SDS.

### Radial Arm Maze (RAM)

#### Animals

Male Sprague–Dawley rats, aged between 12 and 14 weeks, were used in all experiments. They were bred and maintained in cages made of Makrolon and filled with autoclaved woodchips in the Core Unit of Biomedical Research, Division of Laboratory Animal Science and Genetics, Medical University of Vienna. Food and water in bottles was available *ad libitum*. The room was illuminated with artificial light at an intensity of about 200 l× in 2 m from 5 am to 7 pm. Experiments were carried out between 8 am and 2 pm All procedures were carried out according to the guidelines of the Ethics committee, Medical University of Vienna, and were approved by Federal Ministry of Education, Science and Culture, Austria (BMWFW-66.009/0114-WF/II/3b/2014). All efforts were made to minimize animal suffering and to reduce the number of animals used.

Sixty animals were injected intraperitoneally 1, 5, or 10 mg/kg body weight modafinil dissolved in DMSO or DMSO as vehicle and injected 30 min prior to training on each day and were trained or untrained in the RAM. Low dose treatment for CE was published previously (Eagle et al., 2007). The six groups were: three different doses of modafinil (1, 5, and 10 mg/kg) for trained group, one 10 mg/kg modafinil untrained group and two vehicle trained and untrained group. Animals in the vehicle group were injected with DMSO at a dose of 1 ml/kg of body weight (Bartsch et al., 1976).

#### Apparatus

The maze was made out of black plastic and kept at an elevation of 80 cm above the floor in a room with numerous visual cues. The central platform had a diameter of 50 cm with 12 arms (12 cm × 60 cm) projecting radially outward. A plastic cylinder was used to restrict the movement of rats in the center before the start of training. Lifting of the cylinder was controlled by a pulley system from the far end of the room.

#### Procedure

Radial arm maze training was performed as described in Levin et al. (2010) and Timofeeva et al. (2010) with some modification. In brief: rats were handled for 5 days for adaptation (30 min/day/rat) and also to reduce the body weight to 85%. Water was provided *ad libitum* during the training. The amount of food (ssniff Spezialdiäten GmbH) was provided to maintain a lean, healthy body weight of approximately 85% of the free-feeding weight during training. Out of 12 arms, eight arms were baited with a small piece (40 mg) of same food during the training and four remained un-baited. Before the start of the training, rats were given two habituation sessions in which food was placed all over the maze and rats were allowed to explore the maze and eat the food for 5 min. During the training session, the same arms were baited for each rat once at the beginning of each session to assess WM, while the other four arms were always left un-baited in order to test reference memory. The pattern of baited and un-baited arms were consistent throughout testing for each rat but differed among rats. Each trial started by placing the rat onto the central platform, after 10 s the cylinder was lifted slowly and the rat was allowed to enter any arm. The session lasted 8 min or until all eight baited arms were entered-whatever occurred first. The maze was cleaned between the two trials by using 1% incidin (Ecolab GmbH, Austria). Arms were baited only once and a repeated entry into a baited arm was counted as a WME, whereas any entry into an un-baited arm was recorded as a reference memory error. The rats were given 10 training sessions, one training per day. Untrained group rats underwent similar handling, habituation, food restriction, and spent the same time in the RAM as their counterpart trained group rats except there was no food during the trials so that they do not form any memory. The training sessions were recorded with a computerized tracking video camcorder: 1/3 SSAM HR EX VIEW HAD. Six hours after the end of the tenth training animals were deeply anesthetized with CO_2_ and killed by neck dislocation. Brain tissues were quickly removed and hippocampi were rapidly dissected on a cold plate set at 4–6°C and stored at –80°C till further biochemical analysis.

### Blue Native-Polyacrylamide Gel Electrophoresis (BN-PAGE)

Only the 10 mg/kg body weight dose group was used for proteomic studies. Ten whole hippocampi from each of the four groups were run on BN-PAGE followed by immunoblotting with antibodies against the DA receptor and transporter systems, namely D1R, D2R, D3R, pDAT, and DAT.

#### Homogenization, Sample Preparation and Extraction of Membrane Proteins

All procedures were performed at 4°C as published previously (Saroja et al., 2014). The extracted membrane receptor proteins were then aliquoted and stored at -80°C until use. The extracted fractions were used for BN-PAGE.

#### Blue Native-Polyacrylamide Gel Electrophoresis

Membrane pellets from the ultracentrifugation fraction were solubilized in extraction buffer (1.5 M 6-aminocaproic acid, 300 mM Bis–Tris, pH 7.0) and 10% Triton X-100 (stock solution was added at a ratio of 1:4 to achieve final 2% Triton X-100 concentration) with vortexing every 10 min for 1 h. Following solubilization, samples were cleared by centrifugation at 20,000 × *g* for 60 min at 4°C. The protein content was estimated using the BCA protein assay kit (Pierce, Rockford, IL, USA). Seventy microgram of the membrane protein preparation were applied onto gels. 16 mL of BN-PAGE loading buffer [5% (w/v) Coomassie G250 in 750 mM 6-aminocaproic acid] were mixed with 100 μL of the membrane protein preparation and loaded onto the gel. BN-PAGE was performed in a PROTEAN II xi Cell (Bio-Rad, Germany) using 4% stacking and 5–13% separating gel. The BN-PAGE gel buffer contained 500 mM 6-aminocaproic acid, 50 mM Bis–Tris, pH 7.0; the cathode buffer 50 mM Tricine, 15 mM Bis–Tris, 0.05% (w/v) Coomassie G250, pH 7.0 and the anode buffer 50 mM Bis–Tris, pH 7.0. The voltage was set to 50 V for 1 h, 75 V for 6 h, and was increased sequentially to 400 V (maximum current 15 mA/gel, maximum voltage 500 V) until the dye front reached the bottom of the gel (Kang et al., 2008). Native high molecular mass markers were obtained from Invitrogen (Carlsbad, CA, USA).

#### Immunoblotting

Membrane proteins were transferred from BN-PAGE to PVDF membranes. After blocking of membranes for 1 h with 10 % non-fat dry milk in 0.1% TBST (100 mM Tris–HCL, 150 mM NaCl, pH 7.5, 0.1% Tween 20), membranes were incubated with primary antibodies D1R (diluted 1:5000, Abcam-ab78021, Cambridge, UK), D2R (diluted 1:5000, Abcam-ab21218, Cambridge, UK), D3R (diluted 1:5000, Abcam- ab42114, Cambridge, UK), pDAT (diluted 1:5000, DAT Thr^53^, Phosphosolutions-p435-53, Aurora, United States) and DAT (diluted 1:5000, Abcam- ab111468, Cambridge, UK) and detected with horseradish peroxidase-conjugated anti-rabbit IgG (diluted 1:10000, Abcam- ab6721, Cambridge, UK). Membranes were developed with the Bio-Rad Clarity^TM^ Western ECL Substrate. Arbitrary optical densities of immunoreactive bands were measured by the Image J software program^1^. After developing, total protein staining was done on PVDF membranes as previously described for loading control.

### Radio Ligand-Binding Assay

#### Sample Preparation

Hipocampi and cerebral cortices of rats were homogenized in ice cold 50 mM Tris-HCl (pH 7.4–7.5) in a glass/Teflon Potter homogenizer. Suspensions were diluted to 100 times the tissue volume in 35 ml centrifuge tubes and EDTA was added to the final concentration of 3 mM. The homogenates were centrifuged for 10 min at 35000 ×*g*. The resultant pellets were resuspended in same ice cold buffer and centrifuged again. The obtained pellets were resuspended again and incubated for 2 h in a 23°C water bath subsequently followed by a third centrifugation. Membranes were stored as 50 mg aliquots at -80°C until use.

#### [^3^H]SCH 23390 and [^3^H]Raclopride Binding Assay

In order to test the binding potential of modafinil on D1R and D2R the actual dissociation constants of [3H]SCH 23390 and [3H]Raclopride (American Radiolabeled Chemicals Inc.) were determined as published previously (Dewar et al., 1989; Sun et al., 2012). All stock solutions of drugs and radioligands were prepared in a buffer (120 mM NaCl; 50 mM Tris-HCl; 5 mM KCl; pH 7.4–7.5) at room temperature. [^3^H]SCH 23390 and [^3^H]Raclopride ligands (70–90 Ci/mMol) were evaporized to remove ethanol and 20 nM stocks were prepared. Membrane preparations obtained were prewashed by centrifuging at 35000 × *g* (Sorvall, rotor SS 34) for 10 min at room temperature with a buffer (120 mM NaCl; 50 mM Tris-HCl; 5 mM KCl; pH 7.4–7.5). All experiments were carried out in triplicates.

For D1R, membrane preparations were incubated in a buffer (120 mM NaCl; 50 mM Tris-HCl; 5 mM KCl; pH 7.4–7.5) with the addition of 2 nM of [3H] SCH 23390 and 30 nM Ketanserin (Janssen Pharmaceutica, Beerse, Belgium) in water bath at 23°C for 1 h; while for D2R, membrane preparations were incubated in buffer with 2 nM [3H]Raclopride in water bath at 23°C for 1 h. Non-specific binding was determined by adding 10 μM Butaclamol (Ayerst Laboratories, Montreal, QC, Canada) to the incubation. Five concentrations (0,1 nM–300 nM) of non-labeled SCH 23390 (Schering Corp., Bloomfield, NJ, USA) and Raclopride (Sigma–Aldrich–Chemie GmbH, Seinheim, Germany) were used to construct a saturation curve.

#### Filtration

Membranes with bound radioligand were collected after dilution with buffer, with Brandel harvester on glass fiber filters (GF-B or GF-C) pre-soaked in 0.3% polyethylenimine. They were washed three times with buffer and the filters were transferred to picovials. After addition of 1.8 ml toluene scintillation cocktail (50 g PPO and 5 g POPOP dissolved in 10 L toluene), vials were left for 20 min shaking. Radioactivity was counted in the betacounter (Tri-Carb 2100TR, Packard).

### Statistics

Non-linear regression analysis was carried out for reuptake assays to determine the IC_50_ values.

During the training phase, repeated measurement two way ANOVA with the factors, treatment and the training were used. A pairwise multiple comparison was done using the Bonferroni *post hoc* test. Pearson correlation was used for correlation analysis between receptor/transporter complex levels and the WMEs.

For BN-PAGE, densitometry analysis was performed to quantify the level of the receptors and transporters in all four groups. One way ANOVA and Bonferroni *post hoc* analysis was performed to reveal differences. All values were expressed as mean ± SD and the probability level of *p* < 0.05 was considered as statistically significant.

All calculations were performed using GraphPad Prism version 6.00 for Windows, GraphPad Software^2^, San Diego, CA, USA.

For radio ligand-binding assay, dissociation constants KD and maximal number of binding sites BM were estimated by adding five concentrations of unlabeled ligand (SCH 23390 and Raclopride, respectively) and calculating the amount specifically bound ([^3^H]ligand + [^1^H]ligand). Specifically bound ligand was plotted against the ratio ‘bound over free’ (Eadie–Hofstee plot) and the parameters KD (negative slope) and BM (intercept with ordinate) were evaluated by linear correlation.

## Results

### Radial Arm Maze

**FIGURE 2:**
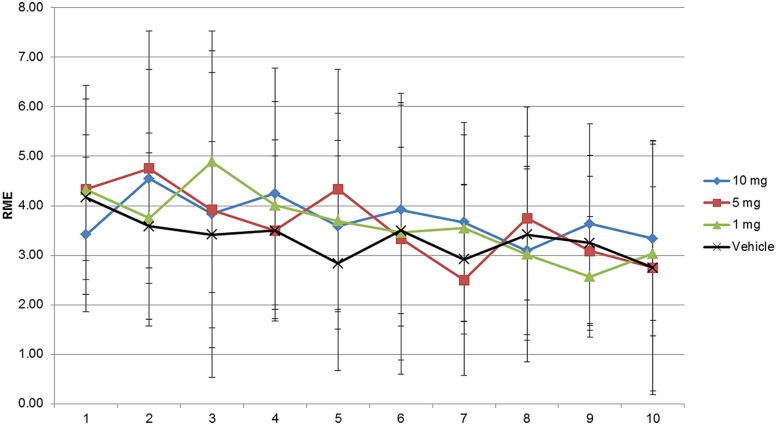
**RME assessment of modafinil in the radial maze for 10 days.** RME’s did not change significantly because of vehicle or modafinil treatment [*F*(3,360) = 0.6625, *p* = 0.5756] or training [*F*(9,360) = 1.658, *p* = 0.0977]. Bonferroni *post hoc* analysis comparing vehicle group with modafinil treated groups at different time points showed no significant changes. Data represented as mean ± SD.

**FIGURE 3:**
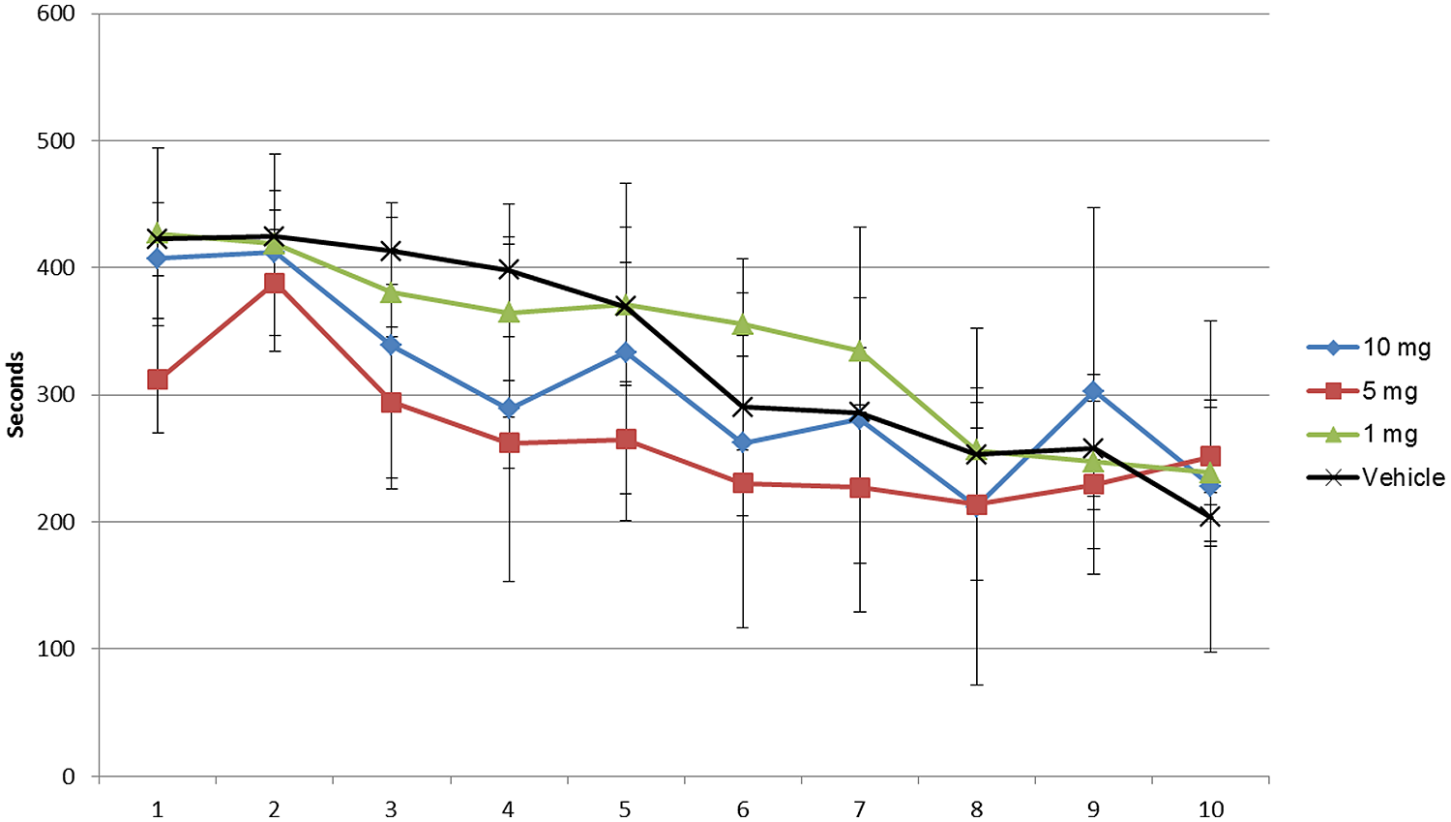
**Latency assessment of modafinil in the radial maze for 10 days.** Latency was significantly different over the training days [*F*(9,360) = 28.65, *p* < 0.0001] and treatment [*F*(3,360) = 18.79, *p* < 0.0001]. Data analyzed using ANOVA and Bonferroni *post hoc* test. Data represented as mean ± SD.

As shown in **Figures 1–3** and Supplementary Tables S1–S3 the animals learned the task and WMEs were gradually decreasing, *F*(9,360) = 17.09, *p* < 0.0001. In the 1 mg/kg body weight dosage group reduction of WME became significant on day 9 while animals administered 5 and 10 mg/kg body weight showed significant reduction of WMEs from day 8, *F*(3,360) = 3.959, *p* = 0.0085. RME’s did not change significantly because of vehicle or modafinil treatment [*F*(3,360) = 0.6625, *p* = 0.5756] or training [*F*(9,360) = 1.658, *p* = 0.0977]. But there was significant difference in the latency’s over the training days [*F*(9,360) = 28.65, *p* < 0.0001] and treatment [*F*(3,360) = 18.79, *p* < 0.0001]. Bonferroni *post hoc* analysis comparing vehicle group with modafinil treated groups at different time points showed significant changes.

### Reuptake Inhibition and Release/Efflux Assays

**FIGURE 4:**
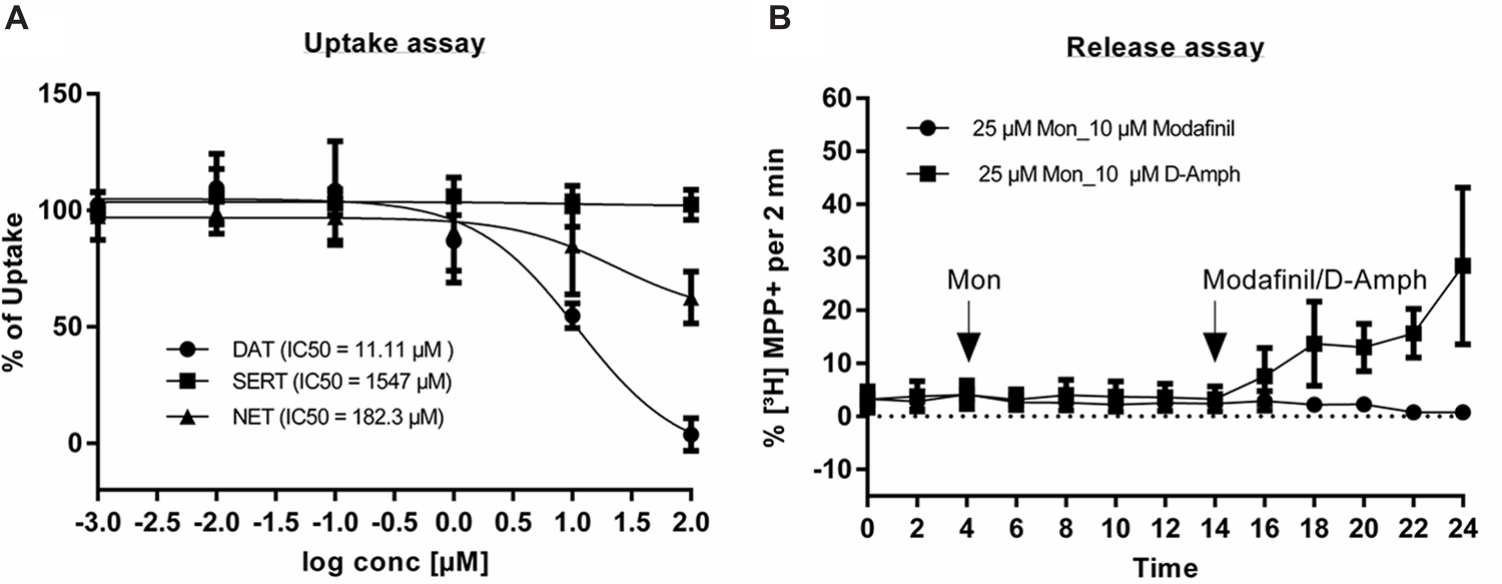
**Substrate inhibition and release assays of modafinil. (A)** For uptake experiments, HEK-DAT, HEK-SERT, and HEK-NET cells were incubated with increasing concentrations of modafinil for 5 min. Subsequently, tritiated substrates were added and the experiments were performed as described in the Section “Materials and Methods.” Unspecific uptake was determined by using 10 μM of mazindole for HEK-DAT and HEK-NET and 10 μM of paroxetine for HEK-SERT. The percentage of maximum uptake was obtained without adding any inhibitory substances. **(B)** The release assay was performed in HEK-DAT cells. Cells were grown on PDL coated coverslips, treated with 0.1 μM ^3^H-MPP^+^ at 37°C for 20 min and washed with KHB buffer for 40 min at superfusion chambers. First three (baseline) and next five fractions were without any compounds and with 25 μM monensin, respectively. Final five fractions were with either 10 μM modafinil or 10 μM amphetamine. Non-linear regression analysis was carried out by using Graphpad prism 6. Values are given as mean ± SEM.

A substrate inhibition assay was performed using HEK293 cells, stably expressing DAT, SERT, and NET. IC_50_ values of modafinil on DAT, SERT, and NET were 11.11 μM, 1547 μM, and 182.3 μM, respectively (**Figure 4A**) suggesting that modafinil rather selectively targets DAT-mediated dopamine uptake as compared to SERT and NET. Subsequently release or efflux assays were performed to examine whether modafinil behaves as a blocker or substrate of DAT. Ten μM of modafinil were used for the release assay, while 10 μM of D-amphetamine served as a positive control, in the presence of 25 μM monensin. DAT-mediated substrate release was not affected by modafinil, indicating that modafinil selectively blocks DAT-mediated uptake, without acting as a substrate (**Figure 4B**).

### Results from BN Followed by Immunoblotting

**FIGURE 5:**
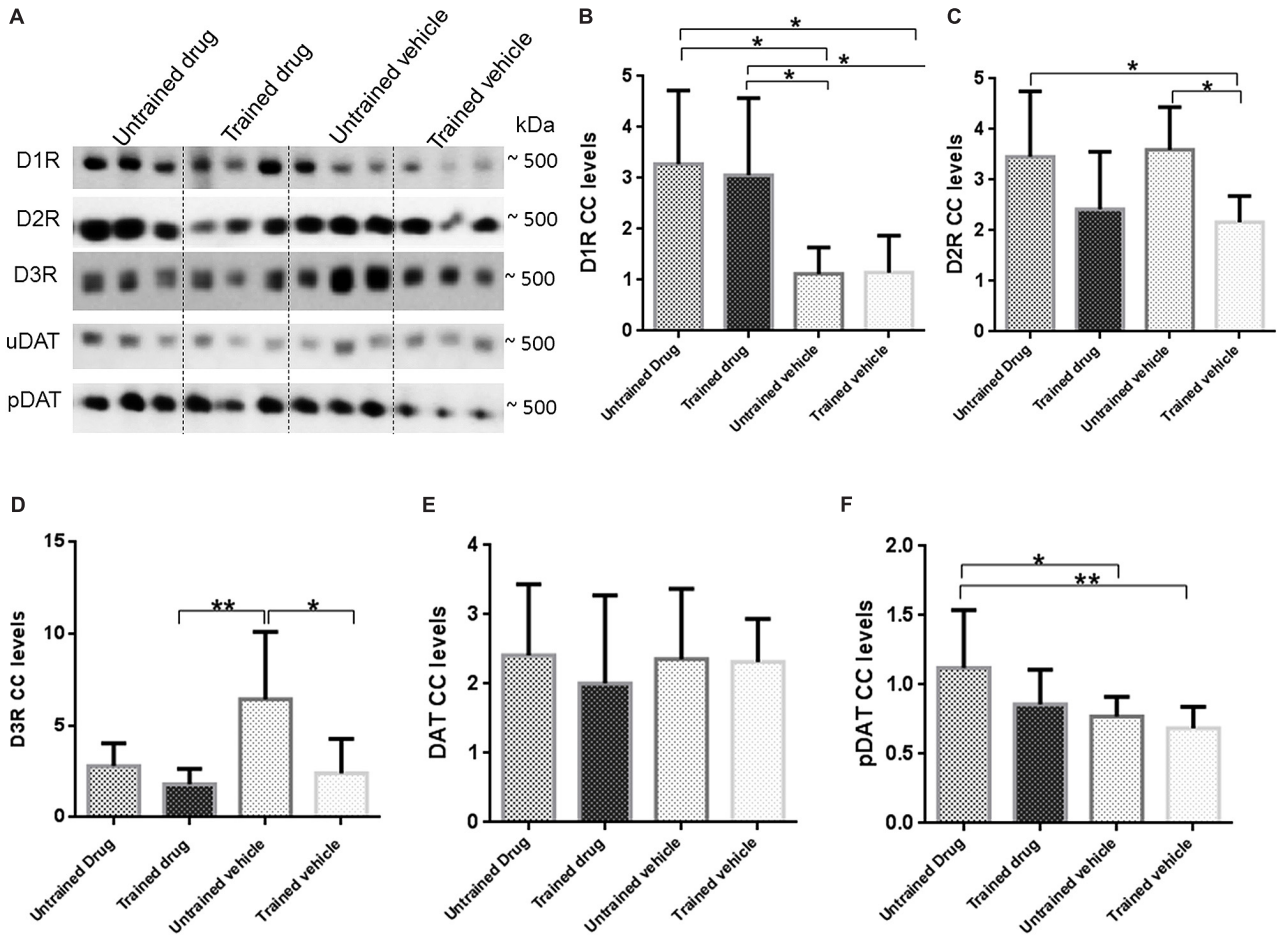
**Representative BN-PAGE western blot images and graphical presentation of D1R, D2R, D3R, DAT, and pDAT levels in untrained drug, trained drug, untrained vehicle and trained vehicle groups.** Total hippocampal membrane fractions were run on 5–13% gradient BN-PAGE gel and blotted onto PVDF membranes. Immunoreactivity of protein CC’s were observed with respective antibodies. Intensity of optical densities of D1R-CC, D2R-CC, D3R-CC, DAT-CC, pDAT-CC were normalized against optical density of the entire corresponding lanes on the membrane. **(A)** Representative images of three bands for dopamine receptor or transporter CC. Approximate molecular weights are indicated; Graphical presentation of receptor and transporters CC levels that were compared are shown as **(B)** D1R (*n* = 10), **(C)** D2R (*n* = 10), **(D)** D3R (*n* = 10), **(E)** DAT (*n* = 10), **(F)** pDAT (*n* = 10). Statistical evaluation was carried out by one way ANOVA followed by *post hoc* Bonferroni test. Data is provided as mean ± SD. (*p < 0.05;**p ≤ 0.01).

In the 10 mg/kg body weight dosage group, hippocampal DA receptor and transporter complexes were separated by BN-PAGE following immunoblotting (**Figure 5A**) and levels were quantified by densitometry analysis. Equal loading was checked by Coomassie R-350 of PVDF membranes as described in Section “Materials and Methods.”

As shown in **Figure 5B**, levels of D1R-CC were increased in both, untrained drug and trained drug in comparison to untrained vehicle, proposing drug effects. D1R-CC levels in trained vehicle showed no significant difference in comparison to untrained vehicle but in trained drug there was an increase in comparison to trained vehicle.

As indicated in **Figure 5C**, D2R-CC levels were decreased in trained vehicle in comparison to untrained vehicle pointing to an effect of WM performance while a trend to decreased levels was observed in trained drug. Untrained drug showed no remarkable differences to untrained vehicle. D2R-CC levels in trained vehicle were decreased in comparison to untrained drug proposing a WM training effect.

D3R-CC levels as demonstrated in **Figure 5D** showed decreased levels in trained drug and trained vehicle as compared with untrained vehicle indicating a WM and drug effect, while a trend to decreased levels was seen in untrained drug.

As shown in **Figures 5E,F**, DAT-CC levels were comparable across groups whereas pDAT-CC levels were increased in untrained drug in comparison with untrained vehicle proposing a drug effect. Trained drug and trained vehicle showed no significant differences to untrained vehicle but trained vehicle was decreased in comparison to untrained drug pointing to a drug effect.

The representative loading control and additional representative full blot is provided in Supplementary Figures S1 and S2.

### Correlations between pDAT, D2R-CC, and WME

The Pearson correlation was calculated to evaluate the relationship between receptor/transporter complex levels and the WMEs on the last day of the training.

**FIGURE 6:**
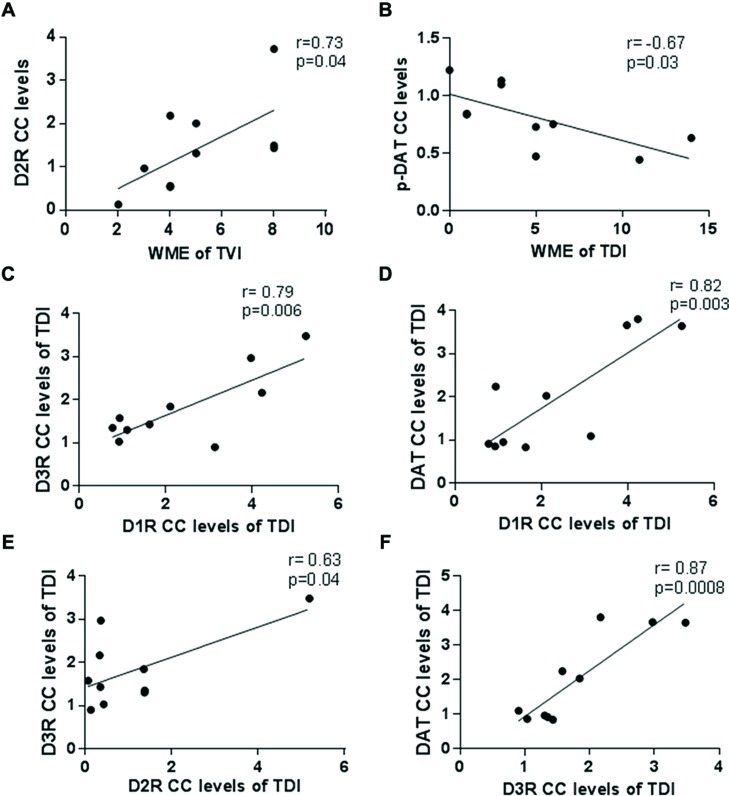
**Correlation analysis data.** The Pearson correlation analysis was calculated to evaluate the link between receptor/transporter CC levels and the WMEs on the last day of training. **(A)** The D2R-CC levels positively correlated with WME’s in the trained vehicle group (*r* = 0.73; *p* = 0.04); **(B)** p-DAT-CC levels negatively correlated with WMEs in the trained drug group (*r* = -0.67; *p* = 0.03); Correlation amongst receptor/receptor and receptor/transporter CC levels was also analyzed by Pearson correlation analysis as **(C)** D3R and D1R in the trained drug group showed a positive correlation (*r* = 0.79; *p* = 0.006); **(D)** DAT and D1R in the trained drug group showed a positive correlation (*r* = 0.82; *p* = 0.003); **(E)** D3R and D2R in the trained drug group showed a positive correlation (*r* = 0.63; *p* = 0.04); **(F)** DAT and D3R in the trained drug group showed a positive correlation (*r* = 0.87; *p* = 0.0008).

As shown in **Figures 6A,B**, D2R-CC levels were positively correlating with WME of trained vehicle (*r* = 0.73; *p* = 0.04) in the RAM whereas levels of pDAT-CC’s were negatively correlating with WME of trained drug (*r* = -0.677; *p* = 0.03).

Moreover, in trained drug, as indicated in **Figures 6C–F**, D1R-CC and D3R-CC (*r* = 0.79; *p* = 0.006); DAT-CC and D1R-CC (*r* = 0.8218; *p* = 0.003); D2R-CC and D3R-CC (*r* = 0.63; *p* = 0.04); DAT-CC and D3R-CC (*r* = 0.87; *p* = 0.0008) showed a positive correlation probably indicating a network or functional interactome of dopamine receptors.

### Radioligand Binding Assay

Since in the BN-PAGE Western blotting, changes of D1-like and D2-like dopamine receptors in the hippocampus after prolonged treatment of rats with modafinil were observed, it was decided to show whether the drug accomplished these effects by direct interaction with these receptors. To our knowledge, modafinil effects have never been tested on these receptors in rat hippocampal tissue, most likely due to their very low densities in that brain region. In pilot experiments (Supplementary Table S4), D1-like receptors at fair densities in the cerebral cortex and hippocampus were observed. However, much lower levels of D2-like receptors were found in these regions. While in the cerebral cortex, saturable [3H]raclopride binding could be resolved, saturation analysis did not result in reliable parameters in hippocampal tissue. Binding of neither of the two radioligands was inhibited to any significant extent by the highest concentration of modafinil (Von Huben et al., 2006) applied.

## Discussion

The major finding of the current study is represented by CE of Sprague–Dawley rats in the RAM by three low or moderate doses of modafinil. Moreover, D2R-CC as well as the phosphorylated-CC, i.e., activated form of the DAT were linked to WMEs.

In contrast to DAT-CC levels, changes of pDAT, D1R, D2R, and D3R-CC were paralleling CE in the RAM by modafinil.

D1R, D2R, and D3R have been already shown to be linked to WM:

D1R antagonism is modulating and linked to WM (Sawaguchi and Goldman-Rakic, 1991, 1994; Von Huben et al., 2006; Beaulieu and Gainetdinov, 2011; Clausen et al., 2011) Furthermore, blocking hippocampal D1 class receptors during learning impaired one trial place memory (Brozoski et al., 1979), and D1R is critically modifying performance accuracy in several WM tasks (Cai and Arnsten, 1997; Henze et al., 2000; Gonzalez-Burgos et al., 2005; Kobori and Dash, 2006; Amico et al., 2007). Finally, the use of previously acquired spatial information in a RAM required D1R activation (Seamans et al., 1998).

In the current study a drug effect by modafinil was observed, however, neither a training effect nor a correlation between D1R-CCs with WMEs was observed. This is not a contradiction, as the current study was carried out determining D1R-CC’s rather than D1R subunits.

D2R-CC’s herein were shown to be reduced by training in the RAM in trained vehicle animals although this was observed as a trend only in trained drug animals. There was, however, a significant positive correlation between D2R-CC’s and WMEs proposing a link between WM and D2R-CC in vehicle-treated rats. And indeed, involvement of the D2R in WM was already suggested (Luciana et al., 1992; Kimberg et al., 1997, 2001; Muller et al., 1998; Mehta et al., 2004): D2R antagonism leading to WM enhancement is consistent with our own observation herein.

As to the D3R, antagonism counteracts cognitive impairment in several rodent and primate procedures including WM (Millan et al., 2010) and Xing et al. (2012) reported that D3R in contrast to D1R does not play a fundamental role in spatial WM.

Herein, training in the RAM reduced D3R-CC’s in vehicle treated animals. This receptor complex correlated with D1R, D2R and DA-CC’s pointing to cooperativity between these dopaminergic systems in WM although no significant correlation between D3R-CC and WMEs was observed.

The DAT-CC levels were comparable between groups, the pDAT-CC levels, however, were reflecting a drug effect. pDAT-CC levels were strongly and negatively correlated with WMEs compatible with a link between this activated DAT form and the WMEs in trained, modafinil-treated rats. Although no significant correlation between DATs and D2R-CC was observed, literature reveals a physical and functional interaction among them (Bowton et al., 2010). This may well indicate that the bands immunoreactive for DAT-CC levels observed in BN-PAGE-WB herein may contain D2R-CC and correlations between DAT-CC, D1R-CC, and D3CC may at least suggest functional if not physical interaction. In addition, other neurotransmitter receptors may be contained in the observed complexes The goal of the study was to identify and quantify D1R, D2R, D3R, DAT-, and pDAT-CC levels and we are aware of the fact that in the observed bands a vast series of individual receptors may be contained (Liu et al., 2006; So et al., 2009; Borroto-Escuela et al., 2014; Fuxe et al., 2014a,b; Khan and Lee, 2014; Kivell et al., 2014).

A putative mechanism of CE in the RAM by modafinil dopamine reuptake inhibition (Elliott et al., 1997; Nail-Boucherie et al., 1998; Mattay et al., 2003; Gerrard and Malcolm, 2007; So et al., 2009) followed by modulation of dopamine receptors may be proposed and a direct effect of modafinil on dopamine receptor complexes was ruled out.

## Conflict of Interest Statement

The authors declare that the research was conducted in the absence of any commercial or financial relationships that could be construed as a potential conflict of interest.

## Footnotes

1 http://imagej.nih.gov/ij/

2 www.graphpad.com

## References

[B1] Amico F. Spowart-Manning L. Anwyl R. Rowan M. J. (2007). Performance- and task-dependent effects of the dopamine D1/D5 receptor agonist SKF 38393 on learning and memory in the rat. *Eur. J. Pharmacol.* 577 71–77. 10.1016/j.ejphar.2007.08.039 17900561

[B2] Bartsch W. Sponer G. Dietmann K. Fuchs G. (1976). Acute toxicity of various solvents in the mouse and rat. LD50 of ethanol, diethylacetamide, dimethylformamide, dimethylsulfoxide, glycerine, N-methylpyrrolidone, polyethylene glycol 400, 1,2-propanediol and Tween 20. *Arzneimittelforschung* 26 1581–1583. 1036956

[B3] Beaulieu J. M. Gainetdinov R. R. (2011). The physiology, signaling, and pharmacology of dopamine receptors. *Pharmacol. Rev.* 63 182–217. 10.1124/pr.110.002642 21303898

[B4] Béracochea D. Cagnard B. Celerier A. le Merrer J. Peres M. Pierard C. (2001). First evidence of a delay-dependent working memory-enhancing effect of modafinil in mice. *Neuroreport* 12 375–378. 10.1097/00001756-200102120-00038 11209953

[B5] Béracochéa D. Celerier A. Peres M. Pierard C. (2003). Enhancement of learning processes following an acute modafinil injection in mice. *Pharmacol. Biochem. Behav.* 76 473–479. 10.1016/j.pbb.2003.09.007 14643846

[B6] Borroto-Escuela D. O. Romero W. Fernandez Narvaez M. Oflijan J. Agnati L. F. Fuxe K. (2014). Hallucinogenic 5-HT2AR agonists LSD and DOI enhance dopamine D2R protomer recognition and signaling of D2-5-HT2A heteroreceptor complexes. *Biochem. Biophys. Res. Commun.* 443 278–284. 10.1016/j.bbrc.2013.11.104 24309097

[B7] Bowton E. Saunders C. Erreger K. Sakrikar D. Matthies H. J. Sen N. (2010). Dysregulation of dopamine transporters via dopamine D2 autoreceptors triggers anomalous dopamine efflux associated with attention-deficit hyperactivity disorder. *J. Neurosci.* 30 6048–6057. 10.1523/JNEUROSCI.5094-09.2010 20427663 PMC2881830

[B8] Brozoski T. J. Brown R. M. Rosvold H. E. Goldman P. S. (1979). Cognitive deficit caused by regional depletion of dopamine in prefrontal cortex of rhesus monkey. *Science* 205 929–932. 10.1126/science.112679 112679

[B9] Cai J. X. Arnsten A. F. (1997). Dose-dependent effects of the dopamine D1 receptor agonists A77636 or SKF81297 on spatial working memory in aged monkeys. *J. Pharmacol. Exp. Ther.* 283 183–189. 9336323

[B10] Chatterjie N. Stables J. P. Wang H. Alexander G. J. (2004). Anti-narcoleptic agent modafinil and its sulfone: a novel facile synthesis and potential anti-epileptic activity. *Neurochem. Res.* 29 1481–1486. 10.1023/B:NERE.0000029559.20581.1a 15260124

[B11] Clausen B. Schachtman T. R. Mark L. T. Reinholdt M. Christoffersen G. R. (2011). Impairments of exploration and memory after systemic or prelimbic D1-receptor antagonism in rats. *Behav. Brain Res.* 223 241–254. 10.1016/j.bbr.2011.03.069 21497169

[B12] Dewar K. M. Montreuil B. Grondin L. Reader T. A. (1989). Dopamine D2 receptors labeled with [3H]raclopride in rat and rabbit brains. Equilibrium binding, kinetics, distribution and selectivity. *J. Pharmacol. Exp. Ther.* 250 696–706. 2527300

[B13] Eagle D. M. Tufft M. R. Goodchild H. L. Robbins T. W. (2007). Differential effects of modafinil and methylphenidate on stop-signal reaction time task performance in the rat, and interactions with the dopamine receptor antagonist cis-flupenthixol. *Psychopharmacology (Berl.)* 192 193–206. 10.1007/s00213-007-0701-7 17277934

[B14] Elliott R. Sahakian B. J. Matthews K. Bannerjea A. Rimmer J. Robbins T. W. (1997). Effects of methylphenidate on spatial working memory and planning in healthy young adults. *Psychopharmacology (Berl.)* 131 196–206. 10.1007/s002130050284 9201809

[B15] Farah M. J. Illes J. Cook-Deegan R. Gardner H. Kandel E. King P. (2004). Neurocognitive enhancement: what can we do and what should we do? *Nat. Rev. Neurosci.* 5 421–425. 10.1038/nrn1390 15100724

[B16] Ferraro L. Antonelli T. O’Connor W. T. Tanganelli S. Rambert F. Fuxe K. (1997). The antinarcoleptic drug modafinil increases glutamate release in thalamic areas and hippocampus. *Neuroreport* 8 2883–2887. 10.1097/00001756-199709080-00016 9376524

[B17] Finke K. Dodds C. M. Bublak P. Regenthal R. Baumann F. Manly T. (2010). Effects of modafinil and methylphenidate on visual attention capacity: a TVA-based study. *Psychopharmacology (Berl.)* 210 317–329. 10.1007/s00213-010-1823-x 20352415

[B18] Fuxe K. Borroto-Escuela D. O. Tarakanov A. O. Romero-Fernandez W. Ferraro L. Tanganelli S. (2014a). Dopamine D2 heteroreceptor complexes and their receptor-receptor interactions in ventral striatum: novel targets for antipsychotic drugs. *Prog. Brain Res.* 211 113–139. 10.1016/B978-0-444-63425-2.00005-2 24968778

[B19] Fuxe K. Tarakanov A. Romero Fernandez W. Ferraro L. Tanganelli S. Filip M. (2014b). Diversity and bias through receptor-receptor interactions in GPCR heteroreceptor complexes. focus on examples from dopamine D2 receptor Heteromerization. *Front. Endocrinol.* 5:71 10.3389/fendo.2014.00071 PMC4026686 24860548

[B20] Gerrard P. Malcolm R. (2007). Mechanisms of modafinil: A review of current research. *Neuropsychiatr. Dis. Treat.* 3 349–364. 19300566 PMC2654794

[B21] Gonzalez-Burgos G. Kroener S. Seamans J. K. Lewis D. A. Barrionuevo G. (2005). Dopaminergic modulation of short-term synaptic plasticity in fast-spiking interneurons of primate dorsolateral prefrontal cortex. *J. Neurophysiol.* 94 4168–4177. 10.1152/jn.00698.2005 16148267

[B22] Henze D. A. Gonzalez-Burgos G. R. Urban N. N. Lewis D. A. Barrionuevo G. (2000). Dopamine increases excitability of pyramidal neurons in primate prefrontal cortex. *J. Neurophysiol.* 84 2799–2809. 11110810 10.1152/jn.2000.84.6.2799

[B23] Hermant J. F. Rambert F. A. Duteil J. (1991). Awakening properties of modafinil: effect on nocturnal activity in monkeys (Macaca mulatta) after acute and repeated administration. *Psychopharmacology (Berl.)* 103 28–32. 10.1007/BF02244069 1672457

[B24] Kang S. U. Fuchs K. Sieghart W. Lubec G. (2008). Gel-based mass spectrometric analysis of recombinant GABA(A) receptor subunits representing strongly hydrophobic transmembrane proteins. *J. Proteome Res.* 7 3498–3506. 10.1021/pr800236u 18563923

[B25] Khan S. S. Lee F. J. (2014). Delineation of domains within the cannabinoid CB1 and dopamine D2 receptors that mediate the formation of the heterodimer complex. *J. Mol. Neurosci.* 53 10–21. 10.1007/s12031-013-0181-7 24264530

[B26] Kimberg D. Y. Aguirre G. K. Lease J. D’Esposito M. (2001). Cortical effects of bromocriptine, a D-2 dopamine receptor agonist, in human subjects, revealed by fMRI. *Hum. Brain Mapp.* 12 246–257. 10.1002/1097-0193(200104)12:4<246::AID-HBM1019>3.0.CO;2-9 11241875 PMC6871975

[B27] Kimberg D. Y. D’Esposito M. Farah M. J. (1997). Effects of bromocriptine on human subjects depend on working memory capacity. *Neuroreport* 8 3581–3585. 10.1097/00001756-199711100-00032 9427330

[B28] Kivell B. Uzelac Z. Sundaramurthy S. Rajamanickam J. Ewald A. Chefer V. (2014). Salvinorin A regulates dopamine transporter function via a kappa opioid receptor and ERK1/2-dependent mechanism. *Neuropharmacology* 86 228–240. 10.1016/j.neuropharm.2014.07.016 25107591 PMC4188751

[B29] Kobori N. Dash P. K. (2006). Reversal of brain injury-induced prefrontal glutamic acid decarboxylase expression and working memory deficits by D1 receptor antagonism. *J. Neurosci.* 26 4236–4246. 10.1523/JNEUROSCI.4687-05.2006 16624944 PMC6673989

[B30] Levin E. D. Timofeeva O. A. Yang L. Petro A. Ryde I. T. Wrench N. (2010). Early postnatal parathion exposure in rats causes sex-selective cognitive impairment and neurotransmitter defects which emerge in aging. *Behav. Brain Res.* 208 319–327. 10.1016/j.bbr.2009.11.007 20015457 PMC2831164

[B31] Liu X. Y. Chu X. P. Mao L. M. Wang M. Lan H. X. Li M. H. (2006). Modulation of D2R-NR2B interactions in response to cocaine. *Neuron* 52 897–909. 10.1016/j.neuron.2006.10.011 17145509

[B32] Luciana M. Depue R. A. Arbisi P. Leon A. (1992). Facilitation of working memory in humans by a d2 dopamine receptor agonist. *J. Cogn. Neurosci.* 4 58–68. 10.1162/jocn.1992.4.1.58 23967857

[B33] Mattay V. S. Goldberg T. E. Fera F. Hariri A. R. Tessitore A. Egan M. F. (2003). Catechol O-methyltransferase val158-met genotype and individual variation in the brain response to amphetamine. *Proc. Natl. Acad. Sci. U.S.A.* 100 6186–6191. 10.1073/pnas.0931309100 12716966 PMC156347

[B34] Mehta M. A. Manes F. F. Magnolfi G. Sahakian B. J. Robbins T. W. (2004). Impaired set-shifting and dissociable effects on tests of spatial working memory following the dopamine D2 receptor antagonist sulpiride in human volunteers. *Psychopharmacology (Berl.)* 176 331–342. 10.1007/s00213-004-1899-2 15114435

[B35] Millan M. J. Buccafusco J. J. Loiseau F. Watson D. J. Decamp E. Fone K. C. (2010). The dopamine D3 receptor antagonist, S33138, counters cognitive impairment in a range of rodent and primate procedures. *Int. J. Neuropsychopharmacol.* 13 1035–1051. 10.1017/S1461145710000775 20663270

[B36] Minzenberg M. J. Yoon J. H. Cheng Y. Carter C. S. (2014). Modafinil effects on middle-frequency oscillatory power during rule selection in schizophrenia. *Neuropsychopharmacology* 39 3018–3026. 10.1038/npp.2014.155 24964814 PMC4229573

[B37] Muller U. Steffenhagen N. Regenthal R. Bublak P. (2004). Effects of modafinil on working memory processes in humans. *Psychopharmacology (Berl.)* 177 161–169. 10.1007/s00213-004-1926-3 15221200

[B38] Muller U. von Cramon D. Y. Pollmann S. (1998). D1- versus D2-receptor modulation of visuospatial working memory in humans. *J. Neurosci.* 18 2720–2728. 9502829 10.1523/JNEUROSCI.18-07-02720.1998 PMC6793089

[B39] Nail-Boucherie K. Dourmap N. Jaffard R. Costentin J. (1998). The specific dopamine uptake inhibitor GBR 12783 improves learning of inhibitory avoidance and increases hippocampal acetylcholine release. *Brain Res. Cogn. Brain Res.* 7 203–205. 10.1016/S0926-6410(98)00023-8 9774732

[B40] Piérard C. Liscia P. Valleau M. Drouet I. Chauveau F. Huart B. (2006). Modafinil-induced modulation of working memory and plasma corticosterone in chronically-stressed mice. *Pharmacol. Biochem. Behav.* 83 1–8. 10.1016/j.pbb.2005.11.018 16439006

[B41] Rebiere F. A. Gerard D. Laurence P. (2010). Process for Enantioselective Synthesis of Single Enantiomers of Modafinil by Asymmetric Oxidation. United. (States) Cephalon France. US 8759583.

[B42] Saroja S. R. Kim E. J. Shanmugasundaram B. Hoger H. Lubec G. (2014). Hippocampal monoamine receptor complex levels linked to spatial memory decline in the aging C57BL/6J. *Behav. Brain Res.* 264 1–8. 10.1016/j.bbr.2014.01.042 24508236

[B43] Sase S. Khan D. Sialana F. Hoger H. Russo-Schlaff N. Lubec G. (2012). Modafinil improves performance in the multiple T-Maze and modifies GluR1, GluR2, D2 and NR1 receptor complex levels in the C57BL/6J mouse. *Amino Acids* 43 2285–2292. 10.1007/s00726-012-1306-y 22614872

[B44] Sawaguchi T. Goldman-Rakic P. S. (1991). D1 dopamine receptors in prefrontal cortex: involvement in working memory. *Science* 251 947–950. 10.1126/science.1825731 1825731

[B45] Sawaguchi T. Goldman-Rakic P. S. (1994). The role of D1-dopamine receptor in working memory: local injections of dopamine antagonists into the prefrontal cortex of rhesus monkeys performing an oculomotor delayed-response task. *J. Neurophysiol.* 71 515–528. 7909839 10.1152/jn.1994.71.2.515

[B46] Scoriels L. Barnett J. H. Soma P. K. Sahakian B. J. Jones P. B. (2012). Effects of modafinil on cognitive functions in first episode psychosis. *Psychopharmacology (Berl.)* 220 249–258. 10.1007/s00213-011-2472-4 21909634

[B47] Scoriels L. Jones P. B. Sahakian B. J. (2013). Modafinil effects on cognition and emotion in schizophrenia and its neurochemical modulation in the brain. *Neuropharmacology* 64 168–184. 10.1016/j.neuropharm.2012.07.011 22820555

[B48] Seamans J. K. Floresco S. B. Phillips A. G. (1998). D1 receptor modulation of hippocampal-prefrontal cortical circuits integrating spatial memory with executive functions in the rat. *J. Neurosci.* 18 1613–1621. 9454866 10.1523/JNEUROSCI.18-04-01613.1998 PMC6792740

[B49] Shuman T. Wood S. C. Anagnostaras S. G. (2009). Modafinil and memory: effects of modafinil on Morris water maze learning and Pavlovian fear conditioning. *Behav. Neurosci.* 123 257–266. 10.1037/a0014366 19331449 PMC2884197

[B50] So C. H. Verma V. Alijaniaram M. Cheng R. Rashid A. J. O’Dowd B. F. (2009). Calcium signaling by dopamine D5 receptor and D5-D2 receptor hetero-oligomers occurs by a mechanism distinct from that for dopamine D1-D2 receptor hetero-oligomers. *Mol. Pharmacol.* 75 843–854. 10.1124/mol.108.051805 19171671 PMC2684927

[B51] Sofuoglu M. DeVito E. E. Waters A. J. Carroll K. M. (2013). Cognitive enhancement as a treatment for drug addictions. *Neuropharmacology* 64 452–463. 10.1016/j.neuropharm.2012.06.021 22735770 PMC3445733

[B52] Sucic S. Dallinger S. Zdrazil B. Weissensteiner R. Jorgensen T. N. Holy M. (2010). The N terminus of monoamine transporters is a lever required for the action of amphetamines. *J. Biol. Chem.* 285 10924–10938. 10.1074/jbc.M109.083154 20118234 PMC2856298

[B53] Sun J. Xu J. Cairns N. J. Perlmutter J. S. Mach R. H. (2012). Dopamine D1, D2, D3 receptors, vesicular monoamine transporter type-2 (VMAT2) and dopamine transporter (DAT) densities in aged human brain. *PLoS ONE* 7:e49483 10.1371/journal.pone.0049483 PMC3504049 23185343

[B54] Timofeeva O. A. Eddins D. Yakel J. L. Blackshear P. J. Levin E. D. (2010). Hippocampal infusions of MARCKS peptides impair memory of rats on the radial-arm maze. *Brain Res.* 1308 147–152. 10.1016/j.brainres.2009.10.040 19854162 PMC2806651

[B55] Turner D. C. Clark L. Dowson J. Robbins T. W. Sahakian B. J. (2004a). Modafinil improves cognition and response inhibition in adult attention-deficit/hyperactivity disorder. *Biol. Psychiatry* 55 1031–1040. 10.1016/j.biopsych.2004.02.008 15121488

[B56] Turner D. C. Clark L. Pomarol-Clotet E. McKenna P. Robbins T. W. Sahakian B. J. (2004b). Modafinil improves cognition and attentional set shifting in patients with chronic schizophrenia. *Neuropsychopharmacology* 29 1363–1373. 10.1038/sj.npp.1300457 15085092

[B57] Von Huben S. N. Davis S. A. Lay C. C. Katner S. N. Crean R. D. Taffe M. A. (2006). Differential contributions of dopaminergic D1- and D2-like receptors to cognitive function in rhesus monkeys. *Psychopharmacology (Berl.)* 188 586–596. 10.1007/s00213-006-0347-x 16538469 PMC2099258

[B58] Wisor J. (2013). Modafinil as a catecholaminergic agent: empirical evidence and unanswered questions. *Front. Neurol.* 4:139 10.3389/fneur.2013.00139 PMC3791559 24109471

[B59] Xing B. Guo J. Meng X. Wei S. G. Li S. B. (2012). The dopamine D1 but not D3 receptor plays a fundamental role in spatial working memory and BDNF expression in prefrontal cortex of mice. *Behav. Brain Res.* 235 36–41. 10.1016/j.bbr.2012.06.035 22776159

