# Supplementary material for: The effect of modafinil on the rat dopamine transporter and dopamine receptors D1–D3 paralleling cognitive enhancement in the radial arm maze
Source: Front Behav Neurosci. 2015 Aug 19;9:215. doi: 10.3389/fnbeh.2015.00215 (PMC4541367; doi:10.3389/fnbeh.2015.00215)
Supplement: Supplementary file 1 [file Table_1.DOCX]

| **Day** | **Vehicle** | | **Modafinil** | | | | | | | | |
| --- | --- | --- | --- | --- | --- | --- | --- | --- | --- | --- | --- |
|  |  |  | 10 mg/kg (n=10) | | | 5 mg/kg (n=10) | | | 1 mg/kg (n=10) | | |
|  | Mean | SD | Mean | SD | p-Value | Mean | SD | p-Value | Mean | SD | p-Value |
| 1 | 10.27 | 3.47 | 10.3 | 2.95 | 0.9828 | 7.67 | 4.64 | 0.1517 | 9.27 | 4.32 | 0.5551 |
| 2 | 10.45 | 5.28 | 8 | 3.33 | 0.2078 | 10 | 5.41 | 0.8455 | 10.11 | 3.75 | 0.8635 |
| 3 | 8.27 | 5.37 | 7.7 | 5.7 | 0.8117 | 6.33 | 4.01 | 0.3491 | 9.64 | 4.89 | 0.5384 |
| 4 | 7.36 | 2.54 | 7.22 | 3.77 | 0.9197 | 5 | 4.47 | 0.1437 | 8.33 | 4.01 | 0.5066 |
| 5 | 5.55 | 2.91 | 5.22 | 2.33 | 0.7721 | 5.08 | 5.79 | 0.8136 | 6 | 2.69 | 0.7092 |
| 6 | 5.73 | 3.41 | 4.18 | 2.09 | 0.2134 | 4.75 | 4.5 | 0.5714 | 5.19 | 2.46 | 0.674 |
| 7 | 5.09 | 2.63 | 4.33 | 2.6 | 0.5033 | 4.51 | 4.62 | 0.722 | 4.33 | 2.55 | 0.4963 |
| 8 | 5.73 | 2.24 | 3.35 | 2.81 | **0.0400** | 3.81 | 2.02 | **0.0477** | 4.55 | 2.01 | 0.2085 |
| 9 | 5.15 | 2.44 | 3.1 | 2.06 | **0.0459** | 3.41 | 1.26 | **0.0490** | 3.27 | 1.57 | **0.0440** |
| 10 | 5.18 | 2.04 | 3 | 2.47 | **0.0354** | 2.78 | 1.38 | **0.0042** | 2.88 | 2.04 | **0.0155** |

Supplementary table 1 : WME‘s of modafinil treated and vehicle treated groups over the ten days training.
