# Supplementary material for: The effect of modafinil on the rat dopamine transporter and dopamine receptors D1–D3 paralleling cognitive enhancement in the radial arm maze
Source: Front Behav Neurosci. 2015 Aug 19;9:215. doi: 10.3389/fnbeh.2015.00215 (PMC4541367; doi:10.3389/fnbeh.2015.00215)
Supplement: Supplementary file 2 [file Table_2.DOCX]

| **Day** | **Vehicle** | | **Modafinil** | | | | | | | | |
| --- | --- | --- | --- | --- | --- | --- | --- | --- | --- | --- | --- |
|  |  |  | 10 mg/kg (n=10) | | | 5 mg/kg (n=10) | | | 1 mg/kg (n=10) | | |
|  | Mean | SD | Mean | SD | p-Value | Mean | SD | p-Value | Mean | SD | p-Value |
| 1 | 4.17 | 1.27 | 3.42 | 1.56 | 0.2306 | 4.33 | 1.83 | 0.8141 | 4.32 | 2.11 | 0.8420 |
| 2 | 3.58 | 1.88 | 4.55 | 2.98 | 0.3721 | 4.75 | 2.01 | 0.1739 | 3.75 | 1.32 | 0.8086 |
| 3 | 3.42 | 1.88 | 3.83 | 3.30 | 0.7241 | 3.92 | 2.78 | 0.6266 | 4.89 | 2.64 | 0.1481 |
| 4 | 3.50 | 1.51 | 4.25 | 2.53 | 0.4085 | 3.50 | 1.83 | > 0.9999 | 4.01 | 2.10 | 0.5206 |
| 5 | 2.83 | 2.17 | 3.58 | 1.73 | 0.3807 | 4.33 | 2.42 | 0.1415 | 3.69 | 2.18 | 0.3648 |
| 6 | 3.50 | 1.68 | 3.92 | 2.35 | 0.6349 | 3.33 | 2.74 | 0.8625 | 3.46 | 2.57 | 0.9660 |
| 7 | 2.92 | 1.51 | 3.67 | 2.02 | 0.3358 | 2.50 | 1.93 | 0.5761 | 3.55 | 1.88 | 0.3965 |
| 8 | 3.42 | 2.57 | 3.09 | 1.70 | 0.7262 | 3.75 | 1.66 | 0.7243 | 3.01 | 1.73 | 0.6654 |
| 9 | 3.25 | 1.76 | 3.64 | 2.01 | 0.6335 | 3.08 | 1.51 | 0.8104 | 2.57 | 1.22 | 0.3048 |
| 10 | 2.75 | 2.56 | 3.33 | 1.97 | 0.5581 | 2.75 | 2.49 | > 0.9999 | 3.04 | 1.35 | 0.7431 |

**Supplementary table 2: RME‘s of modafinil treated and vehicle treated groups over the ten days training.**
