# Supplementary material for: The effect of modafinil on the rat dopamine transporter and dopamine receptors D1–D3 paralleling cognitive enhancement in the radial arm maze
Source: Front Behav Neurosci. 2015 Aug 19;9:215. doi: 10.3389/fnbeh.2015.00215 (PMC4541367; doi:10.3389/fnbeh.2015.00215)
Supplement: Supplementary file 3 [file Table_3.DOCX]

**Supplementary table 3: Latency‘s of modafinil treated and vehicle treated groups over the ten days training**

| **Day** | **Vehicle** | | **Modafinil** | | | | | | | | |
| --- | --- | --- | --- | --- | --- | --- | --- | --- | --- | --- | --- |
|  |  |  | 10 mg/kg | | | 5 mg/kg | | | 1 mg/kg | | |
|  | Mean | SD | Mean | SD | p-Value | Mean | SD | p-Value | Mean | SD | p-Value |
| 1 | 422.42 | 28.48 | 407.15 | 86.94 | 0.5860 | 312.00 | 42.31 | **< 0.0001** | 427.17 | 66.72 | 0.8303 |
| 2 | 424.75 | 35.91 | 412.15 | 77.74 | 0.6309 | 388.35 | 41.64 | **0.0401** | 418.82 | 26.30 | 0.6633 |
| 3 | 413.08 | 26.53 | 338.70 | 112.88 | **0.0460** | 294.05 | 59.11 | **< 0.0001** | 380.27 | 34.57 | **0.0214** |
| 4 | 398.00 | 52.27 | 288.90 | 135.86 | **0.0219** | 262.35 | 19.98 | **< 0.0001** | 364.74 | 53.62 | 0.1563 |
| 5 | 369.50 | 34.34 | 333.60 | 132.93 | 0.3961 | 265.00 | 42.85 | **< 0.0001** | 371.11 | 61.07 | 0.9400 |
| 6 | 290.50 | 56.00 | 262.05 | 145.16 | 0.5510 | 230.50 | 25.69 | **0.0042** | 355.48 | 25.11 | **0.0022** |
| 7 | 286.00 | 51.56 | 280.75 | 151.62 | 0.9145 | 227.05 | 59.33 | **0.0218** | 334.30 | 42.28 | **0.0261** |
| 8 | 253.25 | 40.30 | 211.95 | 140.14 | 0.3588 | 213.90 | 60.04 | 0.0862 | 256.32 | 48.86 | 0.8739 |
| 9 | 258.00 | 37.41 | 302.75 | 144.11 | 0.3307 | 229.50 | 19.38 | **0.0363** | 247.49 | 68.18 | 0.6588 |
| 10 | 203.83 | 19.43 | 228.20 | 130.20 | 0.5461 | 251.70 | 38.45 | **0.0015** | 238.45 | 57.39 | 0.0726 |
