# Supplementary material for: The effect of modafinil on the rat dopamine transporter and dopamine receptors D1–D3 paralleling cognitive enhancement in the radial arm maze
Source: Front Behav Neurosci. 2015 Aug 19;9:215. doi: 10.3389/fnbeh.2015.00215 (PMC4541367; doi:10.3389/fnbeh.2015.00215)
Supplement: Supplementary file 4 [file Table_4.DOCX]

**Supplementary table 4**

**Radioligand binding assay results**

D1-like and D2-like dopamine receptors in rat cerebral cortex and hippocampus as labelled with [^3^H]SCH 23390 and [^3^H]raclopride, respectively. Influence of modafinil

| radioligand (L): | [^3^H]SCH 23390 | | [^3^H]raclopride | |
| --- | --- | --- | --- | --- |
| tissue: | cerebral cortex | hippocampus | cerebral cortex | Hippocampus |
| [L] (nM): | 2.8 | 2.8, 2.9 | 3.2 | 2.9±0.1 |
| specific binding (fMol/mg tissue): | 11.8 (1) | 2.6, 2.2 (2) | 2.3 (1) | 0.54±0.05 (3) |
| nonspecific binding: | 19% | 57%, 58% | 27% | 71±3% |
| K_D_ (nM): | 2.5 (1) | 2.1, 1.9 (2) | 0.8 (1) | n.d. |
| B_M_ (fMol/mg tissue): | 20.7 (1) | 4.1, 4.0 (2) | 2.9 (1) | n.d. |
| 100 µM modafinil: | +4% | -5%, +2% | +4% | -14±56% |

n.d., not detectable (signal too low for reliable saturation analysis); number of experiments in parentheses. Data are presented as Mean ± Standard deviation.
