# Supplementary figures and images for: The effect of modafinil on the rat dopamine transporter and dopamine receptors D1–D3 paralleling cognitive enhancement in the radial arm maze
Source: Front Behav Neurosci. 2015 Aug 19;9:215. doi: 10.3389/fnbeh.2015.00215 (PMC4541367; doi:10.3389/fnbeh.2015.00215)

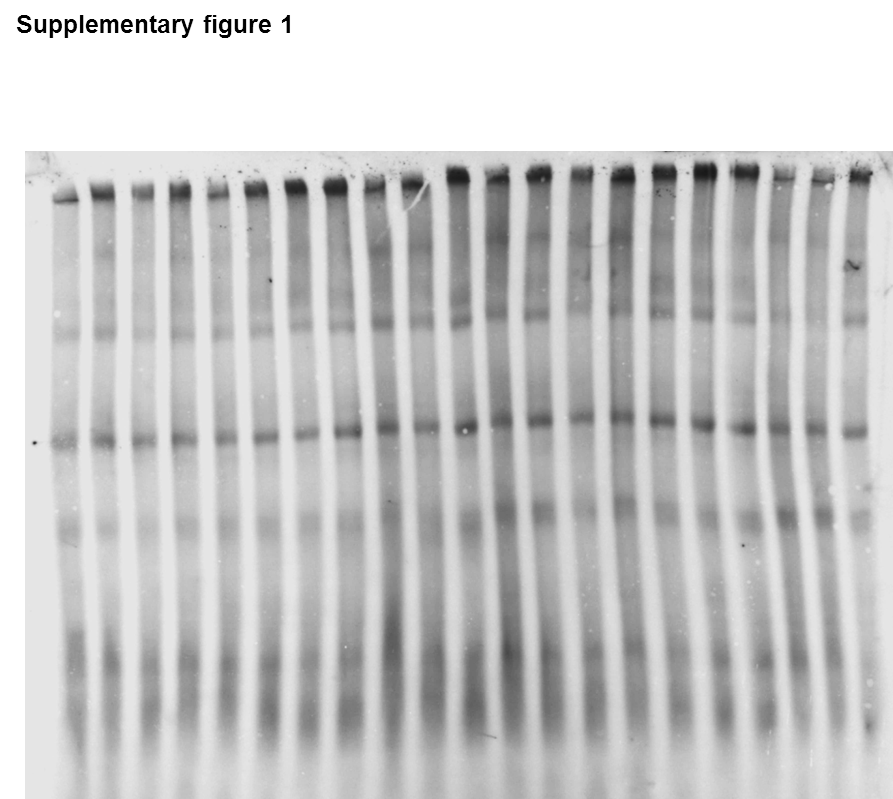

Supplement: Supplementary file 5 [file Image_1.TIF]

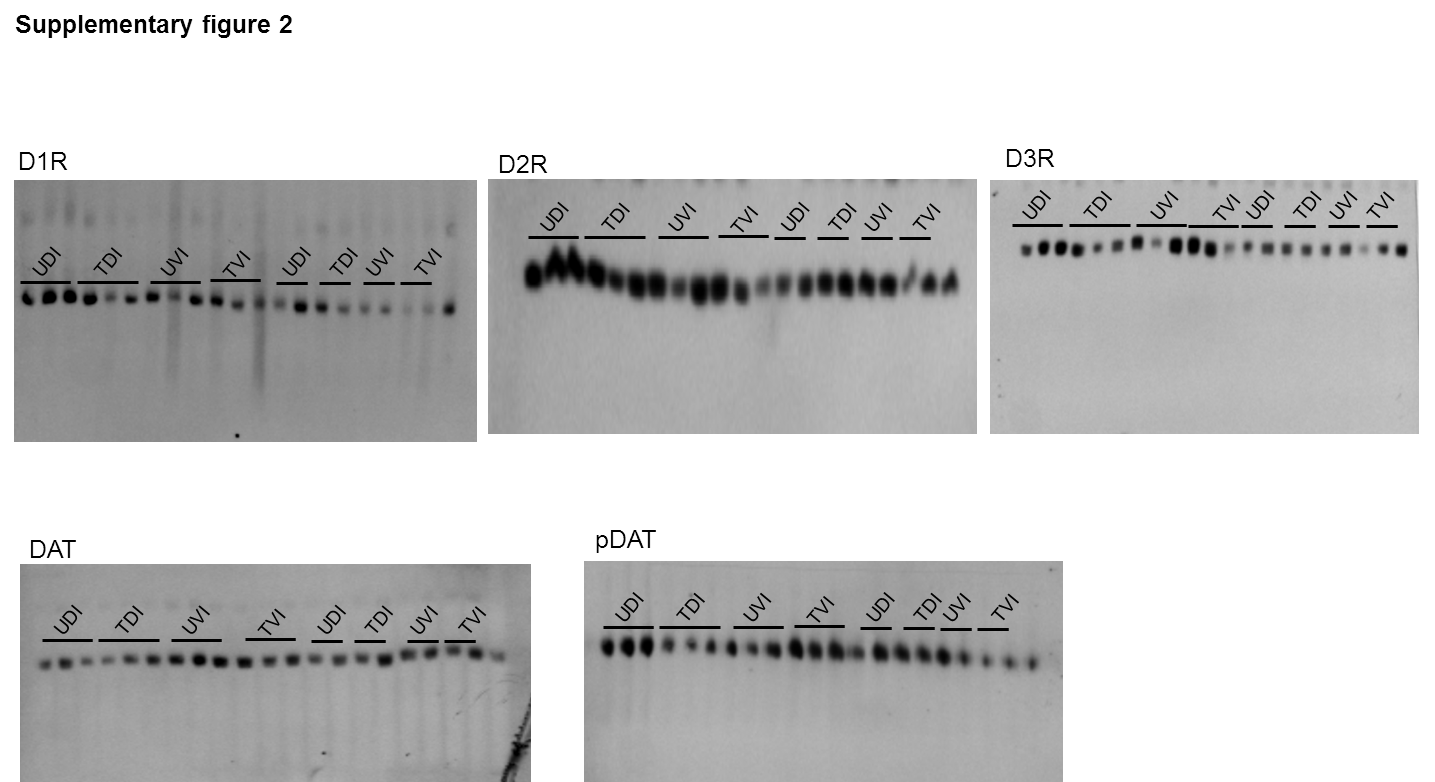

Supplement: Supplementary file 6 [file Image_2.TIF]
